# Pattern of Road Traffic Injuries in Rural Bangladesh: Burden Estimates and Risk Factors

**DOI:** 10.3390/ijerph14111354

**Published:** 2017-11-07

**Authors:** Md. Kamran Ul Baset, Aminur Rahman, Olakunle Alonge, Priyanka Agrawal, Shirin Wadhwaniya, Fazlur Rahman

**Affiliations:** 1 Center for Injury Prevention and Research, Bangladesh, House # B-162, Road # 23, New DOHS, Mohakhali, Dhaka 1206, Bangladesh; kamran@ciprb.org (M.K.U.B.); aminur@ciprb.org (A.R.); fazlur@ciprb.org (F.R.); 2 Department of International Health, Bloomberg School of Public Health, Johns Hopkins University, Baltimore, MD 21205, USA; pagrawa6@jhu.edu (P.A.); swadhwa2@jhu.edu (S.W.)

**Keywords:** road traffic injuries, risk factors, epidemiology, Bangladesh

## Abstract

Globally, road traffic injury (RTI) causes 1.3 million deaths annually. Almost 90% of all RTI deaths occur in low- and middle-income countries. RTI is one of the leading causes of death in Bangladesh; the World Health Organization estimated that it kills over 21,000 people in the country annually. This study describes the current magnitude and risk factors of RTI for different age groups in rural Bangladesh. A household census was carried out in 51 unions of seven sub-districts situated in the north and central part of Bangladesh between June and November 2013, covering 1.2 million individuals. Trained data collectors collected information on fatal and nonfatal RTI events through face-to-face interviews using a set of structured pre-tested questionnaires. The recall periods for fatal and non-fatal RTI were one year and six months, respectively. The mortality and morbidity rates due to RTI were 6.8/100,000 population/year and 889/100,000 populations/six months, respectively. RTI mortality and morbidity rates were significantly higher among males compared to females. Deaths and morbidities due to RTI were highest among those in the 25–64 years age group. A higher proportion of morbidity occurred among vehicle passengers (34%) and pedestrians (18%), and more than one-third of the RTI mortality occurred among pedestrians. Twenty percent of all nonfatal RTIs were classified as severe injuries. RTI is a major public health issue in rural Bangladesh. Immediate attention is needed to reduce preventable deaths and morbidities in rural Bangladesh.

## 1. Introduction

There is a growing consensus in the international health community that road traffic injury (RTI) is a leading cause of death, illness, and disability throughout the world [1,2,3]. According to a World Health Organization (WHO) report, RTI causes 1.3 million deaths per year globally [4]. RTI death rates are more than twice as high in low- and middle-income countries (LMICs) compared to high-income countries (HICs), with almost 90% of all RTI deaths occurring in LMICs [5,6,7,8,9]. In LMICs, RTIs result in losses of up to 5% of the GDP compared with 3% globally [4].

Due to rapid motorization and urbanization in Bangladesh, RTIs are on the rise as in other LMICs, and RTI also represents a leading cause of injury deaths [3,10,11,12,13]. In addition to deaths, RTI is a major cause of hospital admissions at primary and secondary facilities in Bangladesh [14], and traditional data sources such as police data grossly underreport incidence of RTI events in Bangladesh. For example, police statistics showed 3160 deaths due to RTI in 2003, whereas the Bangladesh Health and Injury Survey (BHIS) reported 13,000 RTI deaths in the same year [11]. Similarly, a recent police report showed 2538 deaths due to road crashes in 2012, much lower than the 21,316 road traffic deaths estimated by the WHO [4]. More RTI deaths are recorded in the rural areas of Bangladesh compared to the urban regions [15]. According to the Road Safety Global Report (2015), Bangladesh lacks best practice legislations for all five road safety risk factors, including speeding, helmet use, drink driving, seatbelt use, and child restraint use, which make the situation even worse [4,16].

Bangladesh has had a gradual shift from infectious disease to non-communicable disease and injuries in the past couple of years [11,12,13,17,18,19,20]. The United Nations (UN) declared the period of 2011–2020 as a Decade of Action for Road Safety and two of the 17 Sustainable Development Goals (SDG) indicators aim to reduce global road traffic deaths and injuries by 50% by 2020, in addition to providing access to safe, affordable, accessible, and sustainable transport systems for all by 2030, which is a reflection of the growing recognition of the enormous toll exacted by RTIs [8,21,22]. However, Bangladesh has not taken any remarkable steps to address these unnecessary deaths due to the lack of reliable data on risk factors for RTI [23,24]. To design and implement comprehensive road safety strategies in Bangladesh, knowledge of the magnitude and risk factors for RTI in the country are essential. The objective of this study was to fill the current knowledge gap in RTI epidemiology and risk factors among all populations in rural Bangladesh.

## 2. Methods

“Saving of Lives from Drowning (SoLiD)”, an implementation research study, was conducted between 2013 and 2015 in Bangladesh. In this program, a baseline census was conducted between June–November 2013 in 51 unions of seven sub-districts [25]. The seven sub-districts were Raiganj and Sherpur Sadar in the north of the country, and Matlab North, Matlab South, Daudkandi, Chandpur Sadar, and Manohardi situated in the central part of Bangladesh. The survey covered approximately 1.2 million people in 270,387 households in 993 villages of 51 unions. The survey collected information on all individuals who were residents in the survey areas.

Trained data collectors gathered required information by face-to-face interviews with the heads of households or any household member over 18 years of age who were the most knowledgeable about the household. A repeat visit was made to those households if there was no adult member present or if the respondent was physically or mentally unable to participate in the interview. If an additional second or third visit was unsuccessful, the household was excluded from the census. A set of seven pre-tested structured questionnaires was used to collect relevant information. Data was collected in two stages; the first round collected information on socioeconomic and demographic factors (sex, age, level of education, socioeconomic status), household environment, child and birth history, and health-seeking behavior to understand the household and family status. The survey also collected information on fatal and non-fatal injuries. If a specific injury mortality or morbidity was identified in first round, detailed information was collected regarding the underlying injury mechanisms in the second round. In this study, injury was defined as any external harm resulting from an assault, burn, fall, animal bite, transportation of goods and persons, cuts, poisoning, blunt objects, operating machinery, suffocation, or (near) drowning resulting in the loss of one or more days of normal daily activities, work, or school, and its methodology is described elsewhere [17]. This paper only looked at fatal and non-fatal RTI outcomes. The recall periods for fatal and non-fatal RTIs were one year and six months, respectively.

To ensure the data quality, trained supervisors observed 10% of the conducted interviews and checked 10% of the collected data. They also re-interviewed 2% of the visited households. Field-level research officers also re-checked all data for inconsistencies. If any discrepancy was found, the household was revisited to collect correct information.

A data entry program using SQL Server 2008 was developed and collected data were entered, and then transferred to SPSS version 21 for analysis. All related information of fatal and non-fatal RTIs were retrieved from the primary database for analysis and were de-identified. To analyze the characteristics of RTIs, standard descriptive statistics were used. Description of population by fatal and non-fatal RTIs, sex, age, level of education, socioeconomic status (SES), and sub-district, as well as the place, time, and prior activities of fatal and non-fatal RTIs was provided with proportion. Nonfatal injury events were classified into low, medium, and highly severe injuries based on an index that summarizes indicators such as the number of days an individual required assistance, the number of days lost at work or school, post injury immobility, anatomic and physiologic profile of an injury, post injury hospitalization, surgical treatment, and post injury disability for all events [17]. Fatal RTI rates were calculated per 100,000 population per year and non-fatal RTI rates per 100,000 population per six months. These rates were further analyzed by age, sex, SES, education, and sub-district levels.

Odds of fatal and nonfatal RTI outcomes given independent variables such as age, sex, SES, education, and occupation were assessed using logistic regressions. Results from both bivariate and multivariate logistic regressions are presented. Age was treated as a categorical variable (comprising eight groups: <5 (reference group), 5–9, 10–14, 15–17, 18–24, 25–64, 65+ years). Sex was considered as a binary predictor (reference group was female). Educational (A level and above as a reference group), occupation (agriculture as a reference group), and SES (from lowest to highest) were categorical variables.

### Ethical Statement

Ethical clearance was obtained from the Institutional Review Boards of the Johns Hopkins Bloomberg School of Public Health (approval code: 00004746), International Centre for Diarrheal Disease Research, Bangladesh and the Center for Injury Prevention Research, Bangladesh. For inclusion in the study, informed consent was given by all respondent before they participated.

## 3. Results

### 3.1. Sociodemographic Characteristics of Survey Population

Around 1.2 million people from seven selected sub-districts were covered in the census, and the proportion of the population in each sub-district varied depending upon the number of unions covered for the census. In the census, the proportion of males (48.5%) and females (51.5%) were almost equal. Among the total sample, 39.1% were children (<18 years). A total of 6303 deaths (preceding year) and 119,669 non-fatal injury events (preceding six months) were identified during the census (Table 1). A total of 80 fatal RTIs (8.7% of injury death) and 10,398 non-fatal RTIs (17.8% of injury morbidity) were recorded (Table 2). Of the total, 7.4% of RTIs cases had multiple events.

### 3.2. RTI Mortality and Morbidity

RTI deaths comprised about 1.3% of the deaths that occurred due to any cause in the surveyed population over a recall period of one year. The mortality rate due to RTI was 6.8 (95% CI 55–85) per 100,000 populations per year. The mortality rate was the highest in Raiganj (10.5 per 100,000; 95% CI 5.9–18.9) followed closely by Manohardi (10.3 per 100,000; 95% CI 6.7–15.7).

Across gender profiles, RTI deaths were significantly more in males than females, the mortality rates being 9.2 deaths (95% CI 6.9–12.01) per 100,000 in males compared to 4.7 deaths (95% CI 3.2–6.7) per 100,000 in females. The count of deaths due to RTI was highest in the 25–64 years age group; the unadjusted mortality rate was, however, highest among the elderly age group (14 deaths per 100,000; 95% CI 7.1–26.7) (Table 2). Individuals with no education had the most number of RTI deaths (8.8 per 100,000; 95% CI 6.0–12.9). People with secondary and higher secondary level or higher education had similar mortality rates (Table 2).

The morbidity rate due to RTI was 889 injuries (95% CI 866–900) per 100,000 population per six months. The RTI morbidity rate was highest in Raiganj (1528.4 injuries per 100,000; 95% CI 1456–1605), followed by Manohardi (996.9 injuries per 100,000; 95% CI 954.8–1041 (Table 2). Males suffered significantly higher numbers of injuries than females across all ages, with the morbidity rate being 1551.4 injuries (95% CI 1520–1584) per 100,000 for males versus 264.3 injuries (95% CI 251.7–277.6) per 100,000 for females (Table 2). Adults aged 25 to 64 years sustained the most number of injuries, and also had the highest morbidity rate, 1084.5 injuries (95% CI 1056–1113) per 100,000 population. Adolescents and young adults followed closely, with morbidity rates of 1067.7 injuries (95% CI 989.8–1152) per 100,000 and 1084.5 injuries (1056–1113) per 100,000 population, respectively (Table 2). A higher rate of fatal RTI (8.8/100,000; 95% CI 6.0–12.9) was observed among those who were not educated compared to those with some formal education; however, the differences were not statistically significant (Table 2). In the case of non-fatal RTI events, individuals with higher secondary level and advanced education had significantly higher rates (1279.1/100,000; 95% CI 1195.0–1369.0) of RTI than individuals with lower levels or no education.

With the decrease of the SES index, the rates of fatal RTIs increased with an exception in the high SES quintile, where the rate was found to be the highest (8.9/100,000; 95% CI 5.9–13.5). However, rates of non–fatal RTIs where highest (998.3/100,000; 95% CI 940.7–1017.0) in the highest SES quintile (Table 2).

**Figure 1:**
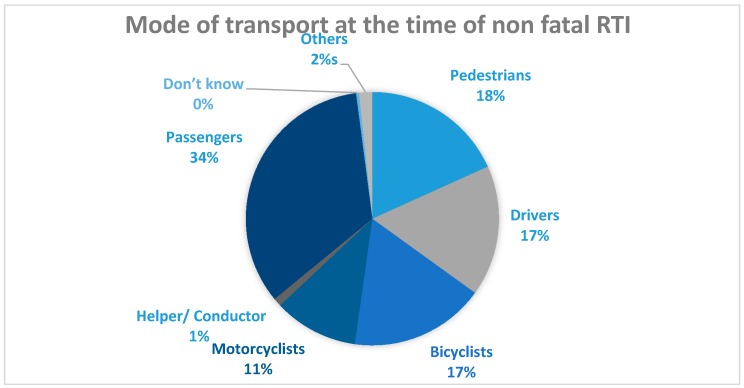
Mode of transport at the time of non-fatal RTI.

**Figure 2:**
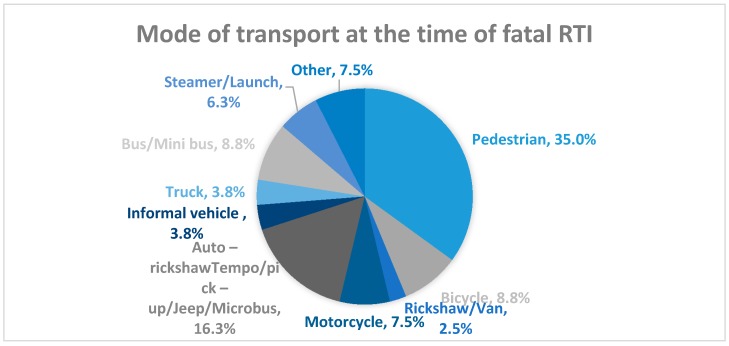
Mode of transport at the time of fatal RTI.

Transport workers had the highest rates for both RTI mortality (46.1/100,000; 95% CI 23.3–90.9) and morbidity (7133.0; 95% CI 6760.0–7525.0) among all occupations (Table 2). When considering the mode of transport, most victims of RTI morbidity were passengers (34%) and pedestrians (18%) (Figure 1). Most RTI mortality, however, occurred among pedestrians (35%). Auto-rickshaw, pickup van, jeep, microbus, bus, bicycle, and motorcycle were the main modes of transportation that an individual was using prior to death resulting from RTI (Figure 2).

**Figure 3:**
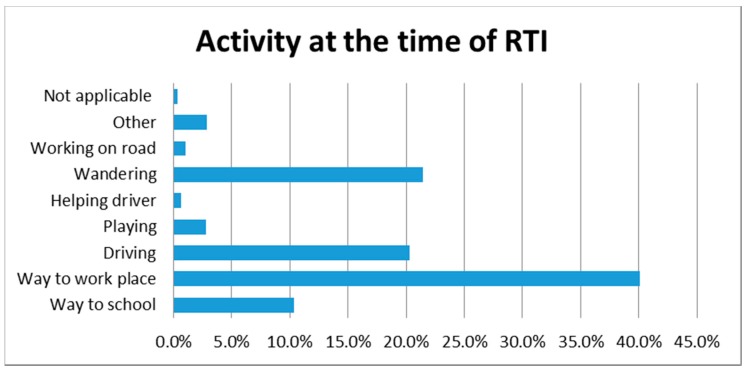
Activity at the time of RTI (both mortality and morbidity).

Around 40.0% of road traffic injuries occurred while an individual was on his way to work, and 21.5% occurred among individuals who were wandering on the streets. One-fifth of the victims were engaged in driving (Figure 3).

**Figure 4:**
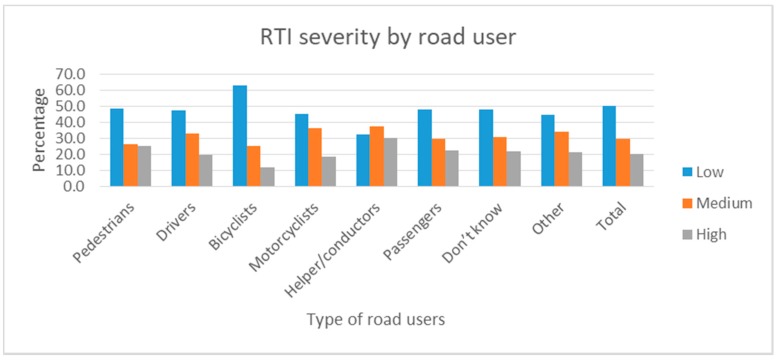
Injury severity index related to road user in RTI morbidity.

The RTI injury severity index showed that 50% of RTI cases had low severity. Almost 20% of cases had been severely injured in a road traffic crash. The highest proportion of high injury severity was found among passengers (37.7%), followed by pedestrians (22.4%) (Figure 4).

The survey findings revealed that 81.4% of motorcyclist RTI victims did not use safety devices (Table 3).

**Figure 5:**
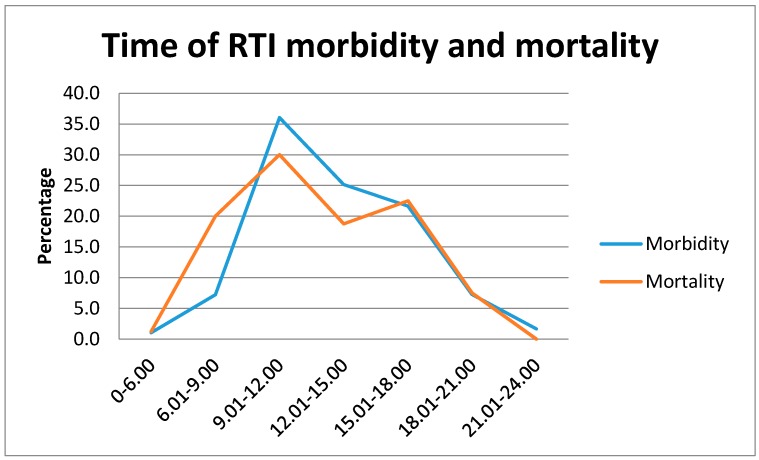
Time of RTI morbidity and mortality.

Most of RTIs happened earlier in the day, between 9:00 a.m. and 12 noon (Figure 5). Most of the respondents (95.4%) mentioned that the injured person was not on drugs. It was also noted that most collisions happened between auto-rickshaws or other informal vehicles (modified vehicles, which have no legal permission to be on the road). Most (46.5%) of the respondents perceived that the road condition was not good.

Multiple logistic regression analysis revealed that males were 4.6 times more at risk of non-fatal RTI (95% CI 4.3–4.9; *p* = 0.000) when compared with females. The risk of a non-fatal road traffic crash increased significantly with increasing age. Individuals aged 15 to 24 years were at the highest risk. Transport workers, such as those driving rickshaws and buses, were 6 times likelier to be in a non-fatal RTI (95% CI 5.5–6.5; *p* = 0.000) than agricultural workers. Education level was not seen to be associated with non-fatal RTI risk. Increasing socioeconomic status was significantly associated with increasing risk of non-fatal RTI (Table 4).

With fatal RTI cases, gender and age were not significantly associated with an increased risk of death due to an RTI. As with non-fatal RTI events, transport workers were 4.5 times more (95% CI 1.8–11.1) at risk than agricultural workers to die in a road traffic accident (Table 4).

## 4. Discussion

The incidence of RTI fatalities was found to be 6.8 deaths (95% CI 55–85) per 100,000 population and non-fatal RTIs were calculated as 889.0 injuries (95% CI 866–900) per 100,000 population. Although RTIs occur in all age groups, the highest rate of fatality (14.0 per 100,000 population) was observed among the older age group (65+ years) followed by 15–17 years (8.1 per 100,000 population), and the highest rate of morbidity was found in the group aged in 24–64 years (1084.4 per 100,000 population). These findings were consistent with other studies from the developing world such as India, Pakistan, Nepal, Vietnam, and Ghana, where RTI was found to be the leading killer and the productive age group was found to be the most likely victim [26,27,28,29,30,31,32]. Other studies also showed that the most active and productive age group, 15–35 years, was the most likely victim of RTI deaths [1,28,33,34,35]. This enhances a serious economic loss to the country, thereby affecting the growth of the county. The reasons behind this trend may be that children have less mobility and are also supervised by adults during road use, but adults have more mobility and exposure to road traffic in order to attend work and studies [36].

Male preponderance in fatal and non-fatal RTIs is concurrent with other studies from Bangladesh and the surrounding countries [7,14,37,38,39]. This is probably because men in Bangladesh have more exposure and movement on the road due to their involvement in work, business, jobs, or studies, whereas females are often restricted to their homes and are responsible for handling household chores [40].

The study findings noticed that no education and lower socioeconomic conditions put individuals at higher risk for road traffic injury deaths. Other studies conducted in LMICs and HICs found similar patterns [11,13,14,36,39,40,41,42,43]. Moreover, the Global Status Report of Road Traffic Injury also projected that poor socioeconomic condition will have a significant role in RTIs and people from a lower socioeconomic status are more likely to be affected [4]. SES is an important predictor for health conditions, especially in RTI situations. RTI is an acute health problem; however, immediate proper health care is not available for poor people in LMICs [4,9,11,15].

In this study, mortality and morbidity were highest among transport workers, such as rickshaw pullers. It has been reported in different studies that more RTIs were seen among students and laborers [14,44,45,46]. The open design of a rickshaw could be one reason why such rickshaw pullers are at higher risk [47]. It is interesting to note that passengers were more involved in non-fatal RTIs, whereas pedestrians were involved in deadly RTIs. Similar findings have been highlighted in many other studies in LMICs [10,15]. Pedestrians appear to be at greater risk of death and injury due to RTI. This could be clarified by the fact that people mostly travel on foot in rural areas of Bangladesh and these areas are not equipped with properly designed roads. This puts pedestrians at a higher risk of being knocked down by motor vehicles [48]. Most of the studies in LMICs showed the same picture of RTIs, in that pedestrian are predominantly affected by road traffic crashes owing to the mixture of slow and fast vehicles in addition to pedestrians on the same roads [49,50,51,52]. It was also noted that most of the collisions happened between auto-rickshaws or other informal three-wheelers, which are the main vehicles used on rural roads [15,35,48].

In the present study, the highest number of crashes happened between morning and noon (9 a.m. to 12 p.m.). This pattern is similar to other studies from Bangladesh and the surrounding regions, such as India, where the highest incidence of RTIs occurred in the same time frame [11,16]. These are the peak hours for traffic on the roads, as children go to school and adults head to their work places [11,15,52]. Motorcyclists who did not use helmets were the most severely injured. Similar findings for the lack of helmet use was also found in other studies [12,17,52].

The strength of this study is that data was collected from a large sample size covering all individuals in the survey area. The information was collected through two-stage verbal interviews by trained data collectors and all data was cross-checked. However, data was collected mostly from rural areas of Bangladesh; therefore, the findings may not be generalizable to urban areas of Bangladesh and these sample households were not nationally representative [4]. However, the topography, road networks, and road structures of rural Bangladesh are similar in nature, thus the study outcomes are generalizable to other areas of rural Bangladesh [15].

Additionally, the study did not collect data on other risk factors such as exposure time, kilometers driven, and knowledge of safety practices. Furthermore, not all respondents were victims or eye witnesses, which gives some limitation to data accuracy.

Other established common risk factors such as lack of awareness, lack of engineering modification, and travelling on overcrowded or poorly maintained vehicles could not be captured in the context of this study.

## 5. Conclusions

The magnitude of fatal and non-fatal RTIs is remarkably high in rural communities of Bangladesh, and the working age group and male population are more at risk. Being a pedestrian or a student were also identified as risk factors for both fatal and non-fatal RTIs. Lower socioeconomic condition and no education were the important risk factors for fatal and non-fatal RTIs.

There is obviously a need for targeted and directed intervention approaches to reduce road traffic injuries. Some examples of interventions include road safety education programs for road users, safe child pedestrian programs through school education, community awareness programs, first responder training at the community level, the implementation of safety measures for non-motorized vehicles, and engineering modifications for speed calming. Such approaches should be directed towards vulnerable road users [15,19,46,51]. Immediate attention should be made to strengthen the intervention measures in an integrated manner to prevent these unexpected events.

## Figures and Tables

**Table 1:** Sociodemographic characteristics of fatal and non-fatal road traffic injury (RTI) outcomes.

Characteristics	Total Population (*n* = 1,169,594)		Characteristics	Total Population (*n* = 1,169,594)	
	*n*	(%)	*n*	*n*	(%)
Population by Upazila			Education		
Matlab North	265,897	(22.7)	No education	295,314	(25.3)
Matlab South	209,772	(17.9)	Primary	407,923	(34.9)
Chadpur Sadar	128,356	(11.0)	Secondary	289,658	(24.8)
Raiganj	104,357	(8.9)	A levels and above	63,873	(5.5)
Sherpur	228,519	(19.5)	Not applicable (Under 5 years)	112,664	(9.6)
Manohardi	204,319	(17.5)	SES quintiles		
Daud Kandi	28,373	(2.4)	Lowest	211,601	(18.1)
			Low	218,695	(18.7)
Sex			Middle	238,371	(20.4)
Male	567,674	(48.5)	High	247,716	(21.2)
Female	601,919	(51.5)	Highest	253,210	(21.7)
Age			Occupation		
<5 years	112,664	(9.6)	Agriculture	104,956	(9.0)
5–9 years	139,728	(12.0)	Business	61,661	(5.3)
10–14 years	142,121	(12.2)	Skilled labor (Professional)	89,151	(7.7)
15–17 years	62,098	(5.3)	Unskilled/domestic (Unskilled)	24,520	(2.1)
18–24 years	133,534	(11.4)	Rickshaw/bus (Transport worker)	17,037	(1.5)
25–64 years	508,059	(43.4)	Student	312,537	(26.7)
65+ years	71,389	(6.1)	Retired/unemployed/housewife	408,583	(34.9)
			Other	150,402	(12.4)

**Table 2:** Fatal and non-fatal RTI rates (per 100,000) by sub-districts, sex, age, level of education, SES index, and occupation.

Characteristics	Fatal Road Traffic Injuries (*n* = 80)		Non-Fatal Road Traffic (*n* = 10,398 Events)	
	*n* = 80	Rate/100,000/Year (95% CI)	*n* = 10,398	Rate/100,000/Six Months (95% CI)
All	80	6.8	10,398	889
Upazila				
Matlab North	15	5.6 (3.4–9.3)	2405	904.5 (869.2–941.2)
Matlab South	9	4.3 (2.3–8.2)	1808	861.9 (823.2–902.4)
Chadpur Sadar	6	4.7 (2.1–10.2)	959	747.1 (701.5–795.7)
Raiganj	11	10.5 (5.9–18.9)	1595	1528.4 (1456.0–1605.0)
Sherpur	16	7.0 (4.3–11.4)	1401	613.1 (581.9–645.9)
Manohardi	21	10.3 (6.7–15.7)	2037	996.9 (954.8–1041.0)
Daudkandi	2	7.1 (1.9–25.7)	193	680.0 (591.0–782.8)
Sex				
Male	52	9.2 (6.9–12.01)	8705	1551.4 (1520.0–1584.0)
Female	28	4.7 (3.2–6.7)	1693	264.3 (251.7–277.6)
Age				
<5 years	5	4.4 (1.9–10.4)	355	315.1 (284.0–349.6)
5–9 years	8	5.7 (2.9–11.3)	1007	720.7 (677.7–766.4)
10–14 years	5	3.5 (1.5–8.2)	1048	737.4 (94.2–783.2)
15–17 years	5	8.1 (3.4–18.9)	663	1067.7 (989.8–1152.0)
18–24 years	9	6.7 (3.5–12.8)	1347	1008.7 (956.5–1064.0)
25–64 years	38	7.5 (5.45–10.3)	5510	1084.5 (1056.0–1113.0)
65+ years	10	14.0 (7.6–25.8)	468	655.6 (599.0–717.5)
Education				
No education	26	8.8 (6.0–12.9)	2708	916.9 (883.2–952.0)
Primary	21	5.2 (3.4–7.9)	3627	889.1 (860.8–918.4)
Secondary	23	7.9 (5.3–11.9)	2891	998.1 (962.5–1035)
Higher secondary and above	5	7.8(3.3–18.3)	817	1279.1 (1195.0–1369.0)
Not applicable (Under 5 years)	5	4.4 (1.9–10.4)	355	315.1 (284.0–349.6)
SES quintiles				
Lowest	18	8.5 (5.4–13.5)	1847	872.9 (834.1–913.4)
Low	13	5.9(3.5–10.2)	2006	917.3 (878.2–942.1)
Middle	14	5.9 (3.5–9.9)	1937	812.6 (777.3–849.4)
High	22	8.9 (5.9–13.5)	2131	863.8 (828.0–901.0)
Highest	13	5.1 (3.0–8.8)	2477	998.3 (940.7–1017.0)
Occupation				
Agriculture	11	10.4 (5.8–18.6)	1301	1231.1 (1166.0–1299.0)
Business	6	9.7 (4.4–21.1)	1282	2066.9 (1958.0–2182.0)
Skilled labor (Professional)	11	12.2 (6.8–21.8)	1533	1698.6 (1616.0–1785.0)
Unskilled/domestic (Unskilled)	3	12.1 (4.1–35.5)	332	1335.3 (1200.0–1486.0)
Rickshaw/bus (Transport worker)	8	46.1 (23.3–90.9)	1239	7133.0 (6760.0–7525.0)
Student	15	4.8 (2.9–7.9)	2439	774.4 (744.4–805.6)
Retired/unemployed/housewife	19	4.6 (3.0–7.2)	1575	383.1 (364.7–402.4)
Other	1	17.2 (3.0–97.1)	45	771.9 (577.4–1031.0)
Not applicable	6	4.1 (1.9–9.0)	634	435.9 (403.4–471.1)

**Table 3:** Use of safety device at the time of RTI among motorcyclists.

Motorcycle user	Use of Safety Device at the Time of RTI among Motorcyclists		
	Used	Not Used	Did Not Know
	*n* (%)	*n* (%)	*n* (%)
Morbidity	222 (10.6)	1832 (87.9)	31 (1.5)
Mortality	1 (25.0)	3 (75.0)	0 (0.0)
Total	223 (17.8)	1835 (81.4)	31 (0.7)

**Table 4:** Association between sociodemographic factors and fatal and non-fatal RTI.

Characteristics	Fatal RTI				Non-Fatal RTI			
	OR (95% CI) Unadjusted	*p* Value	OR (95% CI) Adjusted	*p* Value	OR (95% CI) Unadjusted	*p* Value	OR (95% CI) Adjusted	*p* Value
Sex								
Male	2.0 (1.2–3.1)	0.004	1.3 (0.7–2.6)	0.436	5.9 (5.6–6.2)	0.000	4.6 (4.3–4.9)	0.000
Female	1		1					
Age Groups								
<5 years	1		1					
5–9 years	1.3 (0.4–3.9)	0.655	0.5 (0.1–1.5)	0.217	2.3 (2.0–2.6)	0.000	2.9 (2.4–3.5)	0.000
10–14 years	0.8 (0.2–2.7)	0.713	0.7 (0.2–2.6)	0.207	2.3(2.1–2.6)	0.000	3.0 (2.4–3.6)	0.000
15–17 years	1.8 (0.5–6.3)	0.346	0.5 (0.1–2.0)	0.625	3.4 (3.0–3.9)	0.000	3.8 (3.1–4.6)	0.000
18–24 years	1.5 (0.5–4.5)	0.454	0.5 (0.1–1.8)	0.343	3.2 (2.9–3.6)	0.000	3.7 (3.1–4.5)	0.000
25–64 years	1.7 (0.7–4.3)	0.273	1.3 (0.3–4.8)	0.297	3.4 (3.1–3.8)	0.000	3.7 (3.0–4.5)	0.000
65+ years	3.2 (1.1–9.2)	0.036	0.5 (0.1–1.5)	0.742	2.2 (1.9–2.5)	0.000	2.8 (2.3–3.5)	0.000
Level of education								
Not applicable	0.6 (0.2–2.0)	0.370	0.7 (0.4–1.4)	0.573	0.2 (0.2–0.3)	0.000		
No education	1.1 (0.4–2.9)	0.810	1.3 (0.7–2.5)	0.312	0.7 (0.7–0.8)	0.000	0.9 (0.8–1.0)	0.110
Primary	0.7 (0.2–1.7)	0.400	1.1 (0.3–4.1)	0.475	0.7 (0.6–0.7)	0.000	0.9 (0.9–1.0)	0.118
Secondary	1.0 (0.4–2.7)	0.977	1.0 (0.1–8.2)	0.900	0.8 (0.7–0.8)	0.000	1.0 (0.9–1.1)	0.794
Higher secondary and above	1		1		1		1	
Occupation								
Agriculture	1		1					
Business	0.9 (0.3–2.5)	0.884	1.4 (0.4–2.8)	0.004	1.7 (1.6–1.8)	0.000	1.6 (1.5–1.7)	0.000
Skilled labourer	1.2 (0.5–2.7)	0.702	1.2 (0.5–3.1)	0.969	1.4 (1.3–1.5)	0.000	1.4 (1.3–1.5)	0.000
Unskilled/domestic worker	1.2 (0.3–4.2)	0.812	1.3 (0.4–4.7)	0.636	1.1 (1.0–1.2)	0.184	1.2 (1.1–1.4)	0.004
Transport worker (Rickshaw/bus)	4.5 (1.8–11.1)	0.001	5.1 (2.0–13.0)	0.000	6.2 (5.7–6.7)	0.000	6.0 (5.5–6.5)	0.000
Student	0.5(0.2–1.0)	0.049	0.8 (0.1–1.7)	0.001	0.6(0.6–0.7)	0.000	1.1 (1.0–1.2)	0.067
Retired/unemployed/housewife	0.4 (0.2–0.9)	0.032	0.5 (0.2–1.4)	0.277	0.3 (0.3–0.3)	0.000	1.0 (0.9–1.1)	0.397
Not applicable (children)	0.4 ( 0.1–1.1)	0.068	1.2 (0.0–2.5)	0.201	0.4 (0.3–0.4)	0.000	1.3 (1.1–1.6)	0.000
Not applicable (other)	1.6 (0.2–12.4	0.651	1.2 (0.1–9.9)	0.228	0.6 (0.5–0.8)	0.002	1.2 (0.9–1.7)	0.164
SES index								
Lowest	1							
Low	0.7 (0.3–1.4)	0.325	0.8 (0.4–1.6)	0.681	1.1 (1.0–1.1)	0.132	1.1 (1.0–1.1)	0.067
Middle	0.7 (0.3–1.4)	0.299	0.7 (0.3–1.4)	0.493	0.9 (0.9–1.0)	0.026	0.9 (0.9–1.0)	0.125
High	1.0 (0.6–1.9)	0.892	1.0 (0.5–2.0)	0.289	1.0 (0.9–1.0)	0.654	1.0 (0.9–1.1)	0.855
Highest	0.6 (0.3–1.2)	0.165	0.7 (0.3–1.5)	0.986	1.1 (1.1–1.2)	0.000	1.2 (1.1–1.3)	0.000

## Abbreviations

RTI	road traffic incident
LMIC	low- and middle-income country
HIC	high-income country
CIPRB	Center for Injury Prevention and Research, Bangladesh (CIRPB)
Icddr,b	International Center for Diarrheal Disease Research, Bangladesh (icddr,b)
CI	Confidence Interval
